# Inflammatory Response, a Key Pathophysiological Mechanism of Obesity-Induced Depression

**DOI:** 10.1155/2020/8893892

**Published:** 2020-11-24

**Authors:** Shu-Lin Shao, Da-Qian Liu, Bo Zhang, Shu-Ming Huang

**Affiliations:** ^1^Department of Neuroscience, Institute for Chinese Medicine, Heilongjiang University of Chinese Medicine, Harbin, China; ^2^Department of Orthopedics, The Second Affiliated Hospital of Harbin Medical University, Harbin 150001, China

## Abstract

In recent years, with the acceleration of life rhythm and the increase of social competition, the incidence of obesity and depression has been increasing, which has seriously affected the quality of life and health of people. Obesity and depression, two seemingly unrelated physical and psychological diseases, in fact, are closely related: obese people are more likely to have depression than nonobese ones. We have reviewed and analyzed the relevant research literature and found that the inflammatory response plays a key role in obesity-induced depression. This article will discuss in detail the inflammatory mechanisms by which obesity induces depression.

## 1. Introduction

Obesity is a disease caused by the aberrant accumulation of adipose tissue due to the imbalance between energy absorption and consumption [1], which can increase the incidence of metabolic disease, such as diabetes [2]. Depression is a serious psychiatric illness with a lifetime prevalence up to 20%, whose core symptom is a lack of pleasure [3, 4]. In severe cases, depression may lead to suicide or other negative behaviors [5], making it a major mood disorder that affects people of all ages. Recently, increasing lines of evidence have suggested that obesity increases the peril of developing depression. It has been indicated that high-fat-diet-fed mice will exhibit depressive-like behavior after several weeks' feeding [6, 7]. Additionally, numerous clinical studies have shown that individuals who have high body mass index (BMI) are at significantly increased risk of developing depressive disorder later in life [8, 9].

Although it is generally agreed that obesity is associated with the onset of depression, the precise mechanisms that interactively link these two disease entities remain unclear. To address this issue, we conducted a literature search and analysis of research on experimental and clinical investigations related to depression and obesity. Interestingly, we found that when inflammatory response was assessed, raised levels of inflammatory cytokines could often be detected in the serum of populations and model animals with obesity [10, 11], the same was true of depressed individuals and model animals [12, 13]. Thus, there is a strong reason to believe that inflammation is perhaps a good target to investigate how obesity affects depression. The following is a brief review of the effects of obesity on inflammation and the impacts of the inflammatory response in depression.

## 2. Obesity Induces Elevation of Inflammatory Cytokines

There is growing recognition that obesity links to inflammatory response. Epidemiological studies of individuals with obesity have demonstrated that obese people are usually accompanied by elevated levels of inflammatory cytokines. Nearly 20 years ago, to assess the association between obesity and inflammation, Ziccardi et al. carried out a comparative study on 40 nonobese and 56 obese premenopausal women, and they found in their serum the increased levels of inflammatory cytokines [14], such as tumor necrosis factor- (TNF-) *α* and interleukin- (IL-) 6. More recently, a clinical study [11] on a larger number of people in mixed-sex groups has reported that compared with 83 controls, in the serum, 117 obesity cases have elevated levels of tumor necrosis factor- (TNF-) *α* and interleukin (IL) families or other various inflammatory mediators. Besides, the relationship between obesity and inflammatory factors has been well demonstrated in rodent models. In this regard, it is worth mentioning that the overexpressing of inflammatory cytokines in obese rodents was first discovered by Hotamisligil [15], who put forward the first molecular mechanism: tumor necrosis factor- (TNF-) *α* links obesity with inflammation. Subsequently, experimental studies have shown that the levels of inflammatory cytokines increase in obese rodents [16, 17] and decrease after intervention treatment [18, 19]. And what is more, once an individual becomes obese, the mast cells' response increases, ultimately resulting in releasing massive inflammatory factors related to allergy [20, 21].

## 3. Mechanisms of Obesity Leading to Inflammation

All those above strongly support an association between obesity and inflammation. It has thus become crucial to gain a better understanding of obesity-induced inflammation. As we all know, the imbalance of energy metabolism, either insufficient consumption or excessive intake, contributes to the aberrant accumulation of adipose in the body. Interestingly, the adipose tissue itself is a culprit that leads to inflammation. Adipose tissue can secrete adipokines and inflammatory factors and therefore should not be simply regarded as an energy storage or metabolic organ [22] but as an endocrine organ as well [23]. Adipocytokines, such as leptin and inflammatory cytokines, can mediate the occurrence of inflammation [24, 25].

Apart from the well-known adipocytes, macrophages, predominantly microglia in the brain, also play an integral role in the development of obesity-induced inflammation. It has been proved in experimental research that the migration of macrophages to adipose tissue is increased under obesity [26]. Macrophages usually have two transformed phenotypes, namely M1 macrophages and M2 macrophages [27]. The process through which obesity causes inflammation is largely attributed to M1 polarized macrophages that release proinflammatory cytokines [28]. An experimental study conducted by Kawanishi et al. has suggested that high-fat-diet-induced obesity in mice is accompanied by macrophage infiltration into adipose tissue and that suppression of macrophage infiltration or promotion of M1 to M2 macrophage conversion by experimental methods inhibits the adipose tissue inflammation [29], which has further confirmed the importance of macrophage infiltration, especially of M1 phenotype macrophages, in the process of obesity-induced inflammation.

Although the exact mechanisms responsible for obesity resulting in macrophage infiltration still elude us, there is a hypothesis accepted by most scholars that hypoxia in adipose tissue is one of the possible mechanisms of macrophage infiltration [30]. This hypothesis believes that tissue hypoxia caused by excessive fat accumulation can induce inflammation [31]. Hypoxia in adipose tissue can trigger off the inflammatory behavior of macrophages via the hypoxia-inducible factor (HIF) pathway [32], ultimately leading to inflammatory response. An observational study that used high-fat-diet-induced obese mice with HIF gene knocked out and normal mice as research objects found that the obesity-induced inflammatory response was reduced after HIF gene was knocked out [33]. Furthermore, adipose tissue hypoxia can also activate the inflammation by affecting the function of the endoplasmic reticulum. An experiment carried out by Kawasaki et al. has demonstrated a strong association between endoplasmic reticulum stress and adipose tissue inflammation [34], which suggests that adipose tissue hypoxia can evoke endoplasmic reticulum stress and induce the accumulation of misfolded proteins in the endoplasmic reticulum [35] and eventually activate the inflammatory response through the action of PKR-like endoplasmic reticulum kinase (PERK) and inositol-requiring enzyme- (IRE-) 1, activating transcription factor- (ATF-) 6, and other signals [36].

## 4. Inflammation Resulting in Depression

Extensive research have proved that the inflammatory response is involved in the pathogenesis of various types of depression. Nonetheless, the precise mechanisms of inflammation affecting depression are still not fully elucidated. Over the past decades, the inadequate secretion or dysfunction of the monoaminergic neurotransmitters, including 5-hydroxytryptamine (HT), norepinephrine (NE), and dopamine (DA) [37], has been considered to have the most direct relationship with the onset of depression. This opinion has been attested in numerous clinical and experimental studies [38–40]. Some subsequent studies have reported that there are some shortcomings in the traditional monoaminergic transmitter theory, which make it difficult to explain some phenomena in the research process of depression. For example, antidepressant drugs, which were developed based on this principle and can increase the neurotransmitter of the synaptic space of monoaminergic neurons, can elevate the concentration of monoaminergic neurotransmitters in the synaptic gaps of related neurons in the brain in a short period. However, it takes 1-2 weeks to become effective in clinical practice [41]. Recently, several new hypotheses on the pathogenesis of depression, such as neuronal injury, abnormal neurogenesis, and excessive activation of glial cells, have been put forward and postulated to link with monoaminergic neurotransmitters [42–44].

Neuronal damage, degenerative disorders, or neurogenesis obstacles can consistently induce the deficiencies of these mood-related neurotransmitters [45, 46]. Similarly, the activation of glial cells in the brain may decrease the concentration of monoaminergic neurotransmitters near the synaptic cleft through excessive uptake of the transmitters [47]. Thus, neuronal injury, neurogenesis obstacles, and glial cell overactivation are viewed as potential mechanisms to induce depression [48–50], because they play vital roles in affecting the concentration of monoaminergic transmitters in the synaptic cleft. This viewpoint has been proved in various experimental studies in which animal models of depression replicated by chronic unpredictable mild stimulation (CUMS) were usually accompanied by abnormal neurogenesis, neuronal damage, and glial cell hyperactivation [51–53]. Likewise, these phenomena could be alleviated or reversed by antidepression drug treatment [53–55].

Interestingly, the inflammatory response almost affects all those above mechanisms. Numerous researches have manifested that the inflammatory cytokines can impose effects on monoaminergic neurotransmitters [56–59]. For instance, an experiment conducted by Kaster et al. has shown that the injection of tumor necrosis factor-*α* (TNF-*α*) into the mice may cause a depressive-like behavior [60]. This phenomenon may be induced by the activation of indoleamine 2,3-dioxygenase (IDO) in the peripheral (mainly hepatic), and the conversion of tryptophan (Trp) into kynurenic acid (Kyn), thus affecting the normal secretion of 5-HT [61, 62]. Furthermore, the inflammatory response may cause neurogenesis disorders. This theory has been proved in a related experimental study, in which it has been found that the lipopolysaccharide- (LPS-) induced inflammation in the brain might play a key role in inhibiting the process of neuronal neogenesis from neural stem cells to neurons [63]. When inflammation occurs in the brain, activated M1 phenotype microglia is the main culprit in inhibiting neuronal neogenesis, while M2 phenotype microglia which has anti-inflammatory effect is beneficial to neurogenesis. A study reported that pioglitazone alleviated maternal sleep deprivation induced cognitive deficits in rat offspring by enhancing neurogenesis through switching microglial phenotype from M1 to M2 [64].

Also, the inflammatory reaction may induce neuronal damage. Hoffmann et al. have carried out experimental research, in which the phenomenon of neuronal injury can be observed during Toll-like receptor 2- (TLR2-) mediated neuroinflammation [65]. Besides, the inflammation may play a critical role in stimulating the activation of glial cells. It has been manifested in an experiment report that the synergistic effect of Toll-like receptor 4 (TLR4) and reactive oxygen species (ROS) can mediate the activation of glial cells in lipopolysaccharide- (LPS-) induced inflammatory response, thereby releasing a large number of inflammatory cytokines [66]. An experimental study indicated that inhibiting microglial activation in the brain could alleviate depressive-like behaviors in adolescent mice subjected to maternal separation [67].

It should be noted that obesity may lead to some changes in the permeability of the blood-brain barrier, resulting in obesity-related metabolites and inflammatory factors, such as free fatty acids and IL-6, and even some immune cells, more likely to enter the central nervous system [68]. In obese individuals, not only inflammatory factors but also obesity-related metabolites increase in the blood. In addition to the peripheral inflammatory factors, some peripheral obesity-related metabolites, such as free fatty acid, can also stimulate the central immune cells to release inflammatory factors after they enter the central nervous system [69].

Besides, it should also be added that increasing evidence has shown that obese people often have various degrees of intestinal microbiota imbalance [70–72]. Some dysbacteriosis can induce depression by affecting the metabolism of protein or fat and the secretion of digestive tract hormones and central hormones [73, 74]. Recent studies suggest that animal models implanted with the gut microbiota of depressive people are more likely to be depressed [75, 76]. Moreover, there is emerging data that depression can be treated by regulating gut microbiota [77, 78]. These findings raise the possibility that gut microbiota plays a role in the depression of obesity people with intestinal microbiota imbalance. Yet, it must be pointed out that the causal relationship between obesity and intestinal microbiota is currently uncertain. Although there are a few reports that obesity can change intestinal microbiota, growing studies support the role of gut microbiome in the development of obesity. Intestinal dysbacteriosis can lead to an increase in energy obtained from food or promote energy metabolism to affect energy balance [79, 80], which can inevitably result in obesity. There is ample evidence that regulating intestinal microbiota can reduce the weight of such people [81, 82]. Therefore, in those obesity-induced depression people who have intestinal microbiota imbalance, the intestinal dysbacteriosis may be an initial mechanism through which depression occurs.

Intestinal microbiota imbalance is a complex concept. Many kinds of bacteria parasitize in intestine, and their imbalance does not always induce obesity or depression. Whether the dysbacteriosis (causes obesity or caused by obesity) leads to depression through gut-brain axis needs to be studied further.

The imbalance of intestinal microbiota may influence the function of digestive tract mucosa, which thereby can absorb some small molecules of inflammatory substances, placing the body into a subclinical inflammatory status [83]. Therefore, the imbalance of intestinal microbiota can promote the occurrence of depression through the aforementioned central inflammatory mechanisms, and we cannot ignore the role of inflammation in the process of gut microbiota related depression [84].

## 5. Conclusion

This review took the inflammatory response as an entry point to explore the relationship between obesity and depression. After analyzing recent relevant studies, we presented a new perspective on the occurrence and development of obesity-induced depression: the inflammatory response is a key pathophysiological mechanism of the disorder.

## Additional Points

*Highlights*. (1) Obesity may induce inflammatory response. (2) Some peripheral inflammatory factors can cross the blood-brain barrier and cause inflammatory response in the brain. (3) The cerebral inflammatory response may cause depression through the mechanisms of neuronal injury, excessive activation of glial cells, and hindering neurogenesis.
